# Necessity of external iliac lymph nodes and inguinal nodes radiation in rectal cancer with anal canal involvement

**DOI:** 10.1186/s12885-022-09724-9

**Published:** 2022-06-14

**Authors:** Rong Zheng, YaZhen Zhang, RunFan Chen, Bingjie Guan, YuPing Lin, BiSi Wang, XiaoBo Li, Pan Chi, XiaoPing Chen, BenHua Xu

**Affiliations:** 1 Department of Radiation Oncology, Fujian Medical University Union Hospital, Fuzhou, People’s Republic of China; 2 College of Mathematics and Statistics & FJKLMAA, Fujian Normal University, Fuzhou, People’s Republic of China; 3 Fujian Key Laboratory of Intelligent Imaging and Precision Radiotherapy for Tumors (Fujian Medical University), Fuzhou, People’s Republic of China; 4 Clinical Research Center for Radiology and Radiotherapy of Fujian Province (Digestive,Hematological and Breast Malignancies), Fuzhou, People’s Republic of China; 5 Department of Colorectal Surgery, Fujian Medical University Union Hospital, Fuzhou, People’s Republic of China

**Keywords:** Neoadjuvant radiotherapy, Inguinal lymph nodes, Rectal cancer, Anal canal involvement, Clinical target volume, External iliac lymph nodes, Locally advanced lower rectal cancer

## Abstract

**Background and purpose:**

We aimed to explore the necessity of the external iliac lymph nodes (EIN) along with inguinal nodes (IN) region in clinical target volume (CTV) for rectal carcinomas covering the anal canal region.

**Materials and methods:**

This research premise enrolled 399 patients who had primary low rectal cancer detected below the peritoneal reflection via magnetic resonance imaging (MRI) and were treated with neoadjuvant radiotherapy (NRT), without elective EIN along with IN irradiation. We stratified the patients into two groups based on whether the lower edge of the rectal tumor extended to the anal canal (P group, *n* = 109) or not (Rb group, *n* = 290). Comparison of overall survival (OS), locoregional recurrence-free survival (LRFS), disease-free survival (DFS), as well as distant metastasis-free survival (DMFS) were performed via inverse probability of treatment weighting (IPTW) along with multivariable analyses. We compared the EIN and IN failure rates between the two groups via the Fisher and Gray’s test.

**Results:**

P group showed a similar adjusted proportion along with five-year cumulative rate of EIN failure compared with the Rb group. The adjusted proportion and five-year cumulative rate of IN failure in the P group was higher in comparison to the Rb group. There were no remarkable differences in the adjusted five-year OS, DFS, DMFS or LRFS between the two groups. Anal canal involvement (ACI) exhibited no effect on OS, LRFS, DFS, or DMFS.

**Conclusions:**

During NRT for rectal cancer with ACI, it may be possible to exclude the EIN and IN from the CTV.

## Introduction

Neoadjuvant chemoradiotherapy followed by complete mesorectal excision (TME) is the current standard of treatment for locally advanced rectal cancer [1, 2]. Neoadjuvant chemoradiotherapy, may enhance resectability, sphincter preservation along with local control. Delineating the clinical target volume (CTV), as well as organs at risk (OARs) is a critical step in advanced radiotherapy. The extent of irradiated fields is selected based on pattens of treatment failure, comprising regions with a high relapse while reducing the radiation exposure to adjacent healthy tissues. Nonetheless, the CTV of rectal cancer with anal canal involvement (ACI) includes external iliac lymph nodes (EIN) and inguinal nodes (IN), which is typically the range of the target area of anal cancer. However, clinical evidence to support this target area is lacking.

For rectal cancer with anal canal invasion, elective irradiation of the EIN and IN is recommended [3–6]. However, rather than being based on high-quality research, this guideline is based on expert consensus, and there is no clear proof that EIN and IN failure is prevalent in rectal cancer invading the anal canal. There are some disagreements as to the indications for including these regions for rectal cancer patients [7–10]. Our Center (the Fujian Medical University Union Hospital) commonly does not target the EIN and IN during neoadjuvant radiotherapy (NRT) for locally advanced rectal cancer (LARC) infiltrating the anal canal because of the possible dangers of growing radiation fields, while it is unclear if this is a realistic approach. As a result, we conducted a retrospective analysis to explore the trends of treatment failure for LARC that invaded the anal canal as determined by magnetic resonance imaging (MRI) in patients who were treated with NRT. This data may assist in determining if the current CTV definition is applicable.

Considering that low LARC has a worse prognosis and different recurrence and metastasis patterns compared with middle and upper LARC [11], we assessed the impact of EIN and IN region exclusion from CTV during NRT for low LARC patients with or without ACI on long-term survival outcomes.

## Materials and methods

### Patients

We carried out a retrospective consecutive cohort investigation of patients with primary low LARC (T_3–4_/N_+_) who underwent NRT, without elective EIN and IN irradiation, followed by surgery at our Center between January 2006 and April 2017. Patients were histologically confirmed with rectal adenocarcinoma without distant metastasis. We dined low rectal cancers as below the peritoneal reflection based on pelvic MRI findings. Subjects were stratified into two study groups based on whether the lower edge of the tumor extended to the anal canal (P study group, *n* = 109) or not (Rb study group, *n* = 290).

### Definition of anal canal violation

According to a previous research in Japan, most patients were histologically proved that their bottom edge of the tumor surpassed the anorectal ring when the distance between the lower edge of the tumor and the anal margin was less than 3 cm [12]. The MRI assessment of tumor height was highly reproducible amongst readers, indicating that it may accurately predict dentate line infiltration in low rectal cancer [13]. By employing the intersphincteric groove as the MRI correlate for the anal verge, the average resulted in a tumor height measurement that was remarkably similar to that obtained through endoscopy [14]. Thus, we assessed the tumor's distance from the anal edge and intersphincteric groove on sagittal T2 images in order to determine the rectal tumor's height on MRI. Rectal cancer with a 5 cm gap between the tumor and the anal margin was described as low rectal cancer, while tumor affecting the anal canal with a 3 cm distance was characterized as anal canal cancer.

### Therapeutic schemes

The gross tumor volume (GTV) was contoured using the results of the digital rectal assessment, endoscopy, ultrasonography, along with abdominopelvic magnetic resonance imaging. The CTV was defined as a 5 cm minimum craniocaudal border around the GTV, including the drainage areas of the whole mesorectum, presacral, as well as internal iliac lymph nodes. The GTV and CTV planning target volumes (PTVs) were calculated with an extra margin of 10 mm, respectively. We applied long course radiation (LCRT) to PTV–CTV via 45 Gy in 25 fractions, with or without a simultaneous integrated boost (SIB) to PTV–GTV via 50 Gy in 25 fractions. The dose given to PTV-GTV and PTV-CTV was 25 Gy/5 fractions in short course radiotherapy (SCRT). Patients received pre-operative and postoperative chemotherapy with capecitabine or 5-fluorouracil. All patients received curative total mesorectal excision and sampling/dissection of pelvic nodes 6-8 weeks after pre-operative NRCT.

### Follow-up

Following surgery, patients were evaluated every three months for the first two years, every six months for the following three years, and then yearly thereafter. All patient assessments included a symptoms report, a physical assessment, chest CT, abdominopelvic MRI, as well as testing for six gastrointestinal tumor biomarkers.

### Statistical analyses

Overall survival (OS) was considered as the time period from the start of treatment to the date of death. Disease-free survival (DFS) was considered as the time interval between the start of treatment and the onset of locoregional and/or distant disease failure. The period from the date of initial treatment to the date of extranodal and/or distant lymphatic dissemination was considered as the distant metastasis-free survival (DMFS), and the time period from the date of initial treatment to the date of disease failure in the primary site and/or regional lymph node was regarded as the locoregional recurrence-free survival (LRFS).

After a baseline comparison between the P and Rb groups, an imbalance was found. Subsequently, we utilized the inverse probability weighting (IPTW) approach [15] for component adjustment. The reciprocal of this probability was used as the weight of the individual to establish the correlation model. P-value was utilized to evaluate the balance of covariate distribution prior to and post weighting.

In the present research, the Kaplan Meier approach was utilized to analyze the survival data before and after weighting, and the log-rank test was adopted to analyze whether there were significant statistical differences between the two groups. Subsequently, the weighted Cox proportional hazard model was utilized to determine the IPTW adjusted hazard ratio (HR). We employed the Fisher’s exact tests for statistical analysis on the proportion of EIN and IN failure. The Gray’s test of competitive risk analysis was adopted to analyze the cumulative incidence of EIN and IN failure rate. R 4.0 was utilized to perform the relevant statistical analysis.

## Results

Table 1 summarizes the baseline clinical characteristics of the entire study cohort. Males outnumbered females by a ratio of 1.35:1. The study subjects had a median age of 57 years. Most patients used concurrent chemotherapy (96.0%) and LCRT (95.2%). A total of 73.1% of patients received postoperative chemotherapy. cT1-3, cT4, and cN + were present in 41.6%, 58.4%, and 88.7% of patients, respectively. The median levels of carcinoembryonic antigen (CEA) were 3.30 ng/ml, whilst those of carbohydrate antigen 19–9 (CA199) were 12.62 U/ml. A total of 72.7% of patients were stratified into the Rb group, whereas 27.3% of patients were stratified into the P group. The median follow-up time was 77 months (95% CI 74–84). Clinical features of the two study groups are given in Table 1. After a baseline comparison between the two groups based on whether the anal canal was involved, an imbalance was observed in concurrent chemotherapy regimen (*p* = 0.001), radiotherapy strategies (*p* < 0.001), and number of dissected lymph nodes (*p* = 0.006). After IPTW modification, the prognostic variables were well balanced between the two study groups.

**Table 1:** Clinical characteristics of patients in the unweighted and weighed population

Characteristic	level	Overall	Unweighted population			Weighted population (IPTW)		
			**Rb group, n (%)**	**P group, n (%)**	**p**	**Rb group, %**	**P group, %**	**p**
n		399	290	109				
Gender (%)								
	Female	170 (42.6)	116 (40.0)	54 (49.5)	0.109	42.5	47.1	0.441
	Male	229 (57.4)	174 (60.0)	55 (50.5)		57.5	52.9	
Age								
	< 60	165(41.4)	194 (66.9)	71 (65.1)	0.832	68.3	62.8	0.328
	≥ 60	134(33.6)	96 (33.1)	38 (34.9)		31.7	38.2	
Concurrent chemotherapy regimen (%)								
	Single	232 (58.1)	177 (61.0)	55 (50.5)	0.001	59.5	57.4	0.840
	Combined	143 (35.8)	103 (35.5)	40 (36.7)		35.0	35.6	
	None	24 (6.0)	10 (3.4)	14 (12.8)		5.4	7.0	
Postoperative chemotherapy (%)								
	no	107 (26.9)	70 (24.2)	37 (33.9)	0.068	27.8	32.3	0.414
	Yes	291 (73.1)	219 (75.8)	72 (66.1)		72.2	67.7	
Radiotherapy strategies (%)								
	LCRT	36 (9.0)	26 (9.0)	10 (9.2)	< 0.001	8.7	7.9	0.849
	LCRT + SIB	344 (86.2)	258 (89.0)	86 (78.9)		86.5	85.8	
	SCRT	19 (4.8)	6 (2.1)	13 (11.9)		4.8	6.3	
Number of dissected lymph nodes (%)								
	< 12	195 (48.9)	151 (52.1)	44 (40.4)	0.006	49.6	46.0	0.245
	≥ 12	195 (48.9)	136 (46.9)	59 (54.1)		49.1	49.5	
	none	9 (2.3)	3 (1.0)	6 (5.5)		1.3	4.5	
cT (%)								
	cT1-3	158 (41.6)	122 (44.2)	36 (34.6)	0.115	44.3	34.6	0.107
	cT4	222 (58.4)	154 (55.8)	68 (65.4)		55.7	65.4	
cN (%)								
	cN-	43 (11.3)	30 (10.9)	13 (12.5)	0.790	10.4	11.1	0.844
	cN +	337 (88.7)	246 (89.1)	91 (87.5)		89.6	88.9	
CEA, ng/nl (median [IQR])		3.3 [1.9,7.3]	3.5 [2.0, 7.6]	3.0 [1.7,6.1]	0.252	3.4 [1.9,7.6]	2.8 [1.7, 5.8]	0.191
CA199, U/ml (median [IQR])		12.6 [6.9,22.0]	12.5 [6.6,21.3]	12.8 [7.9,28.2]	0.112	12.7 [6.6, 21.7]	11.9 [7.9,25.8]	0.394

*LCRT* Long course radiotherapy, *SIB* simultaneous integrated boost, *SCRT* short course radiotherapy, *CEA* carcinoembryonic antigen, *CA199* carbohydrate antigen 19–9

**Fig. 1:**
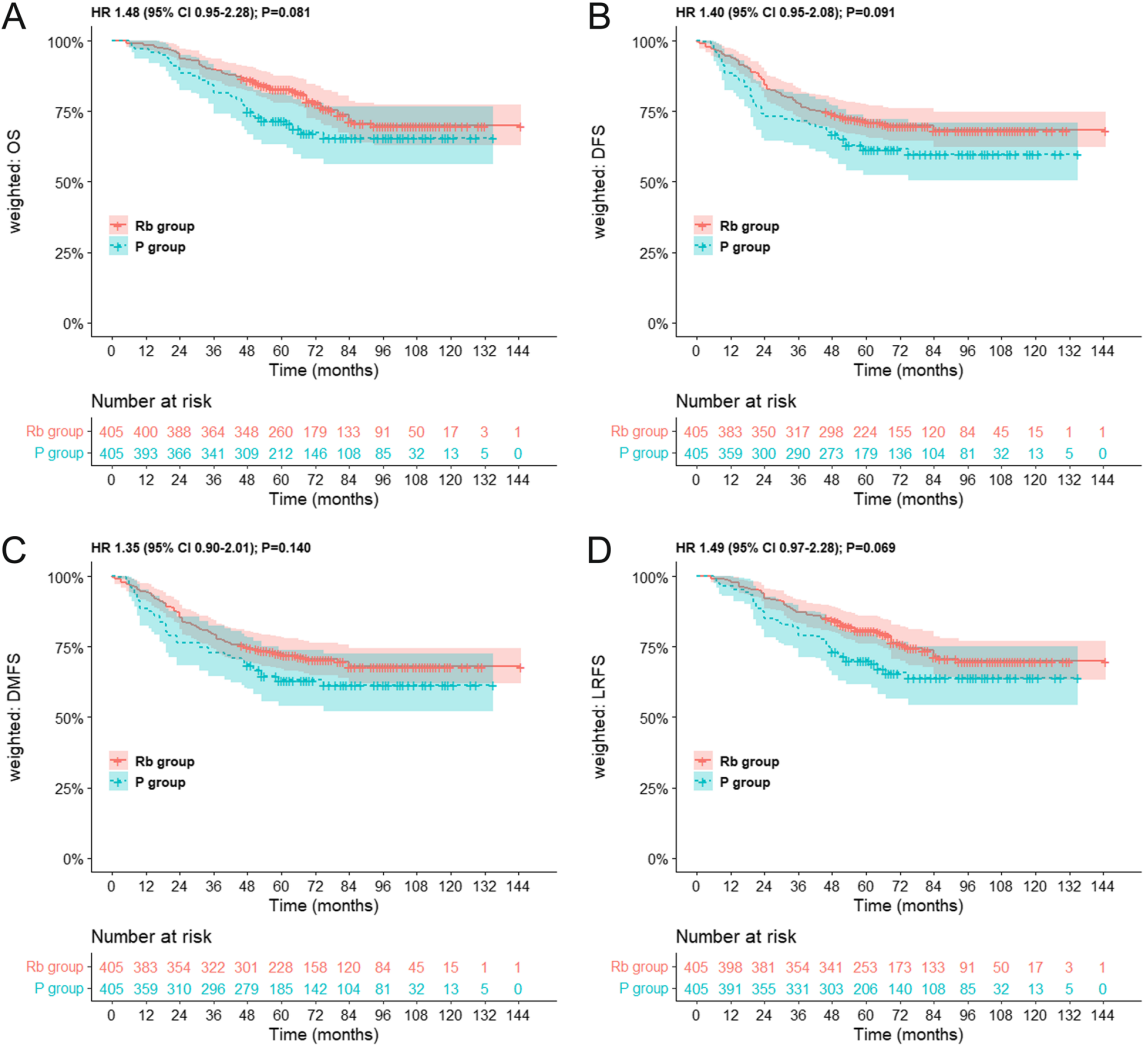
OS, DFS, DMFS, and LRFS stratified by ACI in low LARC patients treated with NRT. OS (A), DFS (B), DMFS (C), and LRFS (D) in the Rb group versus the P group in the entire cohort after IPTW. CI, confidence interval; HR, hazard ratio; IPTW, inverse probability of treatment weighting; OS, overall survival; DFS, disease-free survival; DMFS, distant metastasis free survival; LRFS, locoregional recurrence free survival

We evaluated the effect of anal canal infiltration on the prognosis in low LARC. After IPTW, we found that adjusted five-year OS was 70.7% for P group compared with 82.8% for Rb group (HR 1.48, 95% CI 0.95–2.25, *p* = 0.081; Fig. 1A). The adjusted five-year DFS was 61.5% for the P group versus 71.4% for the Rb group (HR 1.40; 95% CI 0.95–2.08, *p* = 0.091; Fig. 1B). The adjusted five-year DMFS was 63.1% in the P group compared with 72.4% in the Rb (HR 1.35; 95% CI 0.90–2.01, *p* = 0.140; Fig. 1C). The adjusted five-year LRRFS was 69.1% for the P group versus 80.7% for the Rb group (*p* = 0.069, HR 1.49, 95% CI 0.97–2.28; Fig. 1D). Using a multivariate COX model (Table 2), anal canal involvement showed no effect on OS (HR 1.30, 95% CI 0.76–2.22, *P* = 0.336), DFS (HR1.31, 95% CI 0.82–2.08, *P* = 0.260), DMFS (HR 1.23, 95% CI 0.78–1.96, *p* = 0.374), or LRRFS (HR 1.29, 95% CI 0.76–2.19, *p* = 0.346). These results indicated that patients in the P group were more unfavorable, but no remarkable difference was seen between the two study groups. Besides, multivariable analysis exhibited that anal canal invasion was not an independent predictive factor for low LARC patients who received NRT. As for the number of lymph nodes variable, there was a significant difference between none and lymph < 12 in models of OS, DFS, LRFS, and DMFS, and the HRs were much less than 1, implying that patients without lymphatic dissection had a better prognosis. Due to their early pre-operative staging, these patients could opt for local excision without lymph node dissection.

**Table 2:** Multivariable analysis of OS, DMFS, and LRRFS for low LARC patients treated with NRT

variables	OS		DFS		LRFS		DMFS	
	**HR (95% CI)**	**p**	**HR (95% CI)**	**p**	**HR (95% CI)**	**p**	**HR (95% CI)**	**p**
Gender								
Female	Ref		Ref		Ref		Ref	
Male	1.14 (0.66- 1.95)	0.643	1.19 (0.75–1.89)	0.461	1.20 (0.71–2.04)	0.499	1.17 (0.73–1.86)	0.518
Age								
< 60	Ref		Ref		Ref		Ref	
≥ 60	1.52 (0.86–2.67)	0.146	1.50 (0.92–2.44)	0.103	1.67 (0.97–2.88)	0.065	1.39 (0.85–2.28)	0.190
CEA								
≤ 5	Ref		Ref		Ref		Ref	
> 5	1.00 (1.00–1.01)	0.358	1.00 (1.00–1.01)	0.435	1.00 (1.00–1.01)	0.596	1.00 (1.00–1.01)	0.355
CA199								
≤ 37	Ref		Ref		Ref		Ref	
> 37	1.00 (1.00- 1.01)	0.414	1.00 (1.00–1.00)	0.878	1.00 (1.00–1.00)	0.567	1.00 (1.00–1.00)	0.810
Concurrent chemotherapy regimen								
Single	Ref		Ref		Ref		Ref	
Combined	0.89 (0.50- 1.59)	0.702	0.80 (0.49–1.32)	0.386	0.83 (0.47–1.46)	0.517	0.85 (0.51–1.40)	0.523
None	0.29 (0.07- 1.22)	0.092	0.04 (1.4e-4–8.95)	0.237	0.24 (0.49–1.16)	0.076	0.05 (0.00–8.25)	0.249
Postoperative chemotherapy								
No	Ref		Ref		Ref		Ref	
Yes	0.82 (0.42–1.60)	0.559	1.11 (0.61–1.99)	0.741	0.96 (0.49–1.86)	0.897	0.99 (0.55–1.77)	0.972
Number of lymph nodes								
< 12	Ref		Ref		Ref		Ref	
≥ 12	0.94 (0.56–1.60)	0.823	0.92 (0.58–1.46)	0.713	0.92 (0.55–1.53)	0.748	0.96 (0.60–1.55)	0.878
none	1.2e-7 (3.3e-8–4.6e-7)	< 2e-16 ***	1.2e-7 (3.6e-8–3.9e-7)	< 2e-16 ***	1.3e-7 (3.5e-8–4.6e-7)	< 2e-16 ***	1.1e-7 (3.4e-8–3.8e-7)	< 2e-16 ***
Radiotherapy								
LCRT	Ref		Ref		Ref		Ref	
LCRT + SIB	0.70 (0.28–1.77)	0.447	0.81 (0.34–1.89)	0.618	0.76 (0.31–1.86)	0.542	0.77 (0.33–1.80)	0.542
SCRT3	4.77 (0.93–24.52)	0.062	29.33 (0.14–62.55)	0.217	5.54 (0.98–31.49)	0.053	22.37 (0.15–33.03)	0.223
Pathological stage								
ypCR 0	Ref		Ref		Ref		Ref	
ypI	0.49 (0.15–1.55)	0.222	0.95 (0.39–2.33)	0.913	0.65 (0.22–1.90)	0.431	0.79 (0.31–2.01)	0.615
ypII	1.94 (0.81–4.65)	0.138	2.31 (1.07–4.98)	0.033 **	1.95 (0.84–4.55)	0.120	2.34 (1.07–5.11)	0.033 *
ypIII	3.64 (1.59- 8.34)	0.002 **	4.48 (2.15–9334)	6.08e-05 ***	3.83 (1.70–8.61)	0.001 ***	4.32 (2.06–9.09)	0.0001 ***
Anal canal								
Rb group	Ref		Ref		Ref		Ref	
P group	1.30 (0.76–2.22)	0.336	1.31 (0.82–2.08)	0.260	1.29 (0.76–2.19)	0.346	1.23 (0.78–1.96)	0.374

**Fig. 2:**
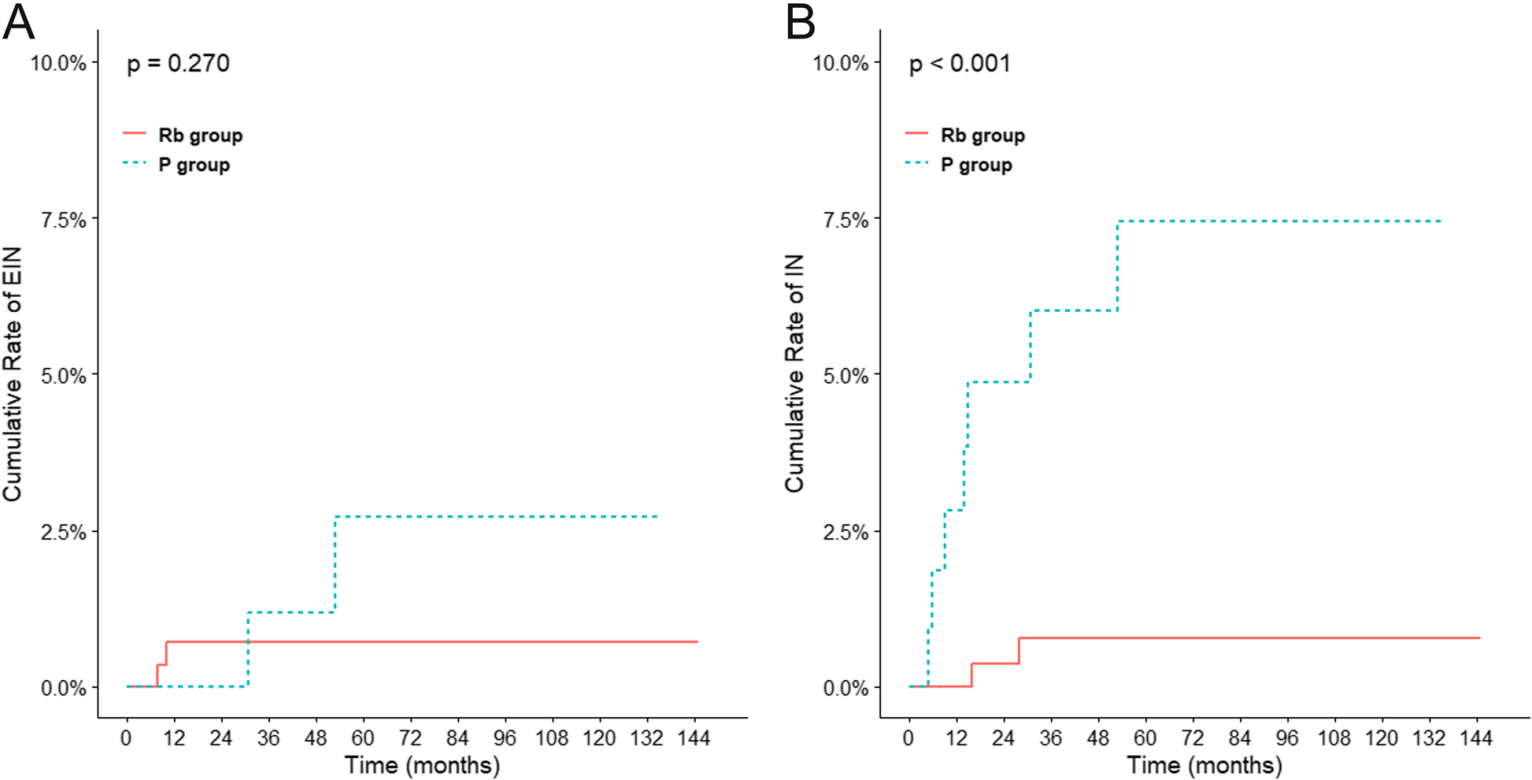
Cumulative incidence of EIN and IN failure stratified by ACI in low LARC patients treated with NRT. Cumulative incidence of EIN (A), and IN (B), in the Rb group versus the P group in the entire cohort after IPTW. EIN, external iliac lymph nodes; IN, inguinal nodes

We further examined the impact of the ACI on EIN and IN failure. The ratios of EIN/IN failure are listed in Table 3. Among the entire cohort, nine (2.3%) of the 399 patients developed IN failure and four (1.0%) developed EIN failure after NRT. The crude IN failure rate in those patients that developed metastasis was significantly higher in P group (7/26, 26.9%) compared with Rb group (2/66, 0.7%; *P* = 0.002). After IPTW, the difference of IN failure rate remained significant in the two groups (6.3% in P group versus 0.7% in Rb group; *P* = 0.008). However, there was no remarkable difference in the EIN failure rate between the two groups before or after weighting. A competitive risk model was used to calculate the cumulative incidence of EIN and IN failure (Fig. 2). P group showed a similar five-year cumulative EIN failure rate comparted with Rb group (3.2% versus 0.7%; *P* = 0.270; Fig. 2A). However, the five-year cumulative IN failure rate in the P group was higher compared with the Rb group (7.4% versus 0.8%; *P* < 0.001; Fig. 2B). These data exhibited that anal canal invasion impacted the risk of IN failure but not the EIN failure in low LARC patients after NRT.

**Table 3:** Proportion of EIN and IN failure stratified by ACI before and after IPTW

	**Unweighted population**			**Weighted population (IPTW)**		
	**Rb group, n (%)**	**P group, n (%)**	**p**	**Rb group, %**	**P group, %**	**p**
Failure	66	26				
EIN (%)	2 (3.0)	2 (7.7)	0.316	2.6	9.0	0.058
IN(%)	2 (3.0)	7 (26.9)	0.002	2.9	26.9	0.000

*IPTW* inverse probability of treatment weighting, *EIN* external iliac lymph nodes, *IN* inguinal nodes

## Discussion

Recently, the definition of rectal cancer CTV has become more personalized. Accurate target volume description is necessary for intensity-modulated radiotherapy (IMRT). Therefore, gaining insights into the failure mode after surgery will help guide the optimization of the irradiation field.

The Radiation Therapy Oncology Group's 2009 consensus report on the definition of the CTV for anorectal cancer was split on whether optional irradiation of the EIN and IN regions in rectal adenocarcinomas with ACI was essential [4]. In addition to the presacral nodes, rectum, mesorectal nodes, obturator nodes, and internal iliac nodes, the 2021 ASTRO Clinical Practice Guideline [6] for radiation therapy for rectal cancer conditionally recommended including the inguinal and external iliac nodes in the CTV for LARC patients with ACI. Additionally, the proposal was made on the basis of expert consensus rather than strong evidence.

Few studies have been conducted to determine the best RT fields for rectal cancer patients treated with ACI. CTV delineation recommendations refer to the characteristics of lymph node metastasis. For tumors that extend to the anal canal, lesions can spread to the EIN and IN [3, 16, 17]. In some retrospective analyses [17, 18], positive EIN were found in 4% to 9% of those subjects with positive lymph nodes. In those patients, the majority of positive lymph nodes stemmed from tumors positioned closer to the anal edge. Maxiaowei Song reported a three-year EIN failure rate of 3.3 percent (7 of 214 patients) [19] in rectal cancer subjects with ACI who were treated with pre-operative or postoperative pelvic RT without including the groin area in the treatment. In the absence of elective irradiation, we observed a very low incidence of EIN metastases in our research. Five-year cumulative EIN failure rates were just 0.7% in the Rb study group and 3.2% in the P study group, and there was no remarkable difference between the two study groups in terms of EIN in metastatic patients.

In rectal cancer, conventional RT fields include regional lymphatics that do not include IN, but standard RT fields in anal cancer include IN due to the increased likelihood of local relapse in this region without elective therapy [4]. Thus, whether RT fields should be expanded to include IN basins for a subgroup of rectal cancer patients with ACI remains uncertain. Publications report a 1% probability of positive inguinal lymph nodes, however all positive INs were identified in rectal tumors that were low-seated [17, 20]. Numerous studies have assessed the IN failure rate in individuals with rectal cancer treated with ACI following pre-operative or post-operative pelvic RT in which the treatment field did not include the groin region; the three-year IN failure rate was 3.7% (8 of 214 patients) [19], the five-year IN failure rate was 4% (6 of 184 patients) [10], and 3.5% (7 of 189 patients) [21]. In our cohort, the proportion of IN failure rate was remarkably higher in metastatic patients with anal canal invasion than those without. However, the IN failure rate was still low in both groups. The five-year cumulative IN failure rate was only 0.8% in the Rb group and 7.4% in the P group, respectively.

Although the risk of the IN failure in the two groups are different, the salvage of IN metastasis appeared feasible, anal canal invasion has no significant influence on the OS, DFS, DMFS, or LRFS with survival analysis and Cox model analysis. Due to the fact that enlarging the RT field to include the groin region is linked with diverse acute and late morbidities, including desquamation and fibrosis of the inguinal skin, wound and bowel complications, extremity edema, as well as femoral neck fractures [10], our findings illustrated that routine elective EIN/IN irradiation for anal canal invasion may be unnecessary. For individuals with ACI who have a low LARC, more effective treatments should be investigated to target the systemic disease instead of ILN irradiation for localized management.

The current research involves a number of limitations. The power along with the generalizability of a small single-center study were limited. The difference in the external iliac node failure between the weighted groups was marginally significant (*p* = 0.058). The difference may reach statistical significance if the sample size is larger enough. The number of dissected lymph nodes is less than twelve in almost half of the patients, and the less than twelve of number of dissected lymph nodes is an independent predictor of poor prognosis in our cohort. Insufficient lymphadenectomy will strongly influence the accuracy of the nodal staging. Since this retrospective analysis may have been skewed by selection bias, prospective multicenter randomized controlled clinical studies are necessary to validate the potential advantages of bypassing the EIN and IN irradiation during NRT.

## Conclusion

We found there was no impact on long-term survival outcomes of EIN and IN region exclusion from CTV during NRT for low LARC patients with or without ACI. The failure rate of EIN and IN was low in LARC patients without elective EIN and IN irradiation, even in patients with ACI. Thus, during NRT for LARC patients, the EIN and IN areas may be exempted from CTV, thus reducing radiation-associated adverse events. Given the small number of patients with relapse in our study, further research is required to determine if the CTV's cranial border may be limited.

## Data Availability

The datasets used and analysed during the current study available from the corresponding author on reasonable request.
